# Usefulness of semi-quantification of ischemic myocardium after adenosine stress magnetic resonance

**DOI:** 10.1186/1532-429X-15-S1-E58

**Published:** 2013-01-30

**Authors:** Lorenzo Monti, Marco Tramarin, Margherita Calcagnino, Veronica Lisignoli, Barbara Nardi, Luca Balzarini

**Affiliations:** 1 Cardiology, I.R.C.C.S. Istituto Clinico Humanitas, Rozzano(MI), Italy; 2 Radiology, I.R.C.C.S. Istituto Clinico Humanitas, Rozzano (MI), Italy

## Background

Adenosine stress MR shows high sensitivity and intermediate-to-high specificity; therefore, there is a number of false positive patients that is referred to useless coronary angiography studies after a positive adenosine stress MR. We sought to verify whether the (semi) quantification of the amount of ischemic myocardium can improve the management of patients after a positive adenosine stress MR study. According to previous nuclear medicine evidence, we defined a 10% LV mass cut-off for the diagnosis of clinically relevant ischemia.

## Methods

80 MR studies were classified as positive or negative according to the presence or absence of a reversible perfusion defect ("old approach"). We re-analyzed the perfusion studies dividing each one of the 16 AHA segment of the 3 perfusion slices in 2 similar sub-segments ( subepicardial and subendocardial): each one of the resulting 32 segment represent about 3% of the myocardial mass. We re-defined ("new approach") as ischemic the patients with > 3 subsegments (>10% of LV mass) with reversible lesions, and as non-ischemic the patients with 3 or less positive subsegments. Patients were classified as affected by clinically relevant ischemia if the post-adenosineMR coronary angiography confirmed coronary lesions > 50%, and non-affected if the angio was negative and/or no MACE were observed after a > 6 months follow-up.

## Results

Mean age 60.7 years old. Mean follow-up = 18 months (5-37). Pre-test prevalence of CHD: 64%. Prevalence of clinically relevant ischemia = 17.5%. With the old approach we obtained a sensitivity and specificity of 92.9% and 72.7 respectively. With the new approach we obtained a sensitivity and specificity of 85.7% and 90.9% respectively. Global accuracy of the stress MR exam increased from 76.2 to 90%.

## Conclusions

A visual semi-quantification of the ischemic myocardium improves patient management after a positive adenosine stress MR study.

## Funding

None

